# Construction of a high-quality yeast two-hybrid (Y2H) library and its application in identification of interacting proteins with key vernalization regulator *Ta*VRN-A1 in wheat

**DOI:** 10.1186/1756-0500-6-81

**Published:** 2013-03-05

**Authors:** Shuanghe Cao, Liuling Yan

**Affiliations:** 1 Department of Plant and Soil Sciences, Oklahoma State University, Stillwater, OK, 74078, USA

**Keywords:** Y2H library, Vernalization, *Ta*VRN-A1, Wheat

## Abstract

**Background:**

Low temperature is required for the competence of winter wheat to flowering (vernalization), and several key components in the vernalization-mediated flowering pathway have been isolated. A Y2H library is a very useful platform to further unravel novel regulators in the flowering pathway. Thus, there is a necessity to construct a high-quality Y2H library using vernalized winter wheat plants.

**Result:**

We described the construction of a high-quality Y2H library using winter wheat plants with cold-treatment for different weeks to maximize pooling interacting proteins during vernalization. The resultant Y2H library contained ~2.5×10^6^ independent clones, with a cell density of ~2.6×10^8^ and an average insert size of ~ 1.5 kb. *Ta*VRN-A1 was used as a “bait” to test the quality of the Y2H library. As a result, several cDNA clones encoding *Ta*SOC1 and *Ta*SVP1 that were known to have a direct binding with *Ta*VRN-A1 were identified, demonstrating that the Y2H screen system constructed in this study was highly efficient. Additional proteins that were discovered but not characterized in previous studies could be novel partners of *Ta*VRN-A1 in wheat.

**Conclusion:**

We established a high-efficient Y2H screen system using the Matchmaker™ technology with several modifications in the critical steps. Ultimately, we provided a successful example to fast and economically create high-quality Y2H libraries for studies on protein interaction in hexaploid wheat.

## Background

Wheat (*Triticum aestivum*, 2n=6×=42, AABBDD) is one of the most important food crops, but the researches on wheat functional genomics lag far behind most of the other crops, such as rice and maize, because of the large size and complexity of its three homoeologous genomes [1]. Along with the application of high-throughput and powerful sequencing technologies, researchers have made great progresses on sequencing wheat genome (http://www.cerealsdb.uk.net/). The post-genomic era is expected to focus on a critical area: identification of multiple functional genes in the same pathway controlling important but complex traits in crops.

Protein interaction networks are of fundamental importance to almost all biological processes, a powerful and high-throughput protein-protein Y2H screen system becomes more and more crucial to identify or mine the partners of proteins in the regulatory complexes [2]. The Y2H screening system is designed especially for high-throughput identification of protein-protein interaction, *i.e.* one against all preys in some developmental processes. In addition to two hybrid system, structure- based software and database have been established to in silicon predict protein-protein interactions in some model species, e.g. Arabidopsis (http://www.arabidopsis.org/portals/proteome/proteinInteract.jsp). Tandem affinity purification (TAP) method is another high-throughput method [3]. Recently, high-throughput BiFC-based screening system has been established to identify target interactions in plant [4]. However, the Y2H library screening is still the most practical tool in high-throughput identification of protein-protein interaction. Several key wheat vernalization genes, *VRN1*, *VRN2*, and *VRN3* responsible for spring-winter growth habit, have been successively cloned and characterized using a map-based cloning approach [5-7], and these genes are good entry points to further unravel the regulatory network of the vernalization pathway using the Y2H screen system.

There are at least two common approaches to construct Y2H libraries. One is the traditional approach in which digestion-ligation procedures are required. At present, this approach is used in few laboratories because of limits in techniques, such as ligation-required, significant size bias, restriction enzyme site-dependent. The other is the prevailing recombination-based approach. Compared to digestion-ligation construction of cDNA library, recombination-based approach only needs one step to incorporate template into vector, which has several merits: simplified directional cloning, improved cloning efficiency, and easy operability. In addition, recombinase-mediated site-specific recombination can reduce chimeric clones and self-ligated vectors. The combination-based approach includes two strategies to create the Y2H library: *in vivo* and *in vitro*. Currently, Gateway® system is the representative of *in vitro* recombination, whereas Matchmaker™ is the exemplary technology of *in vivo* recombination. Gateway® system is powerful and can create an entry cDNA library but it is too laborious and expensive if only to construct a Y2H library [8,9]. Conversely, *in vivo* recombination system can directly create Y2H libraries with less time and effort.

In this study, we used the *in vivo* recombination strategy to generate a high-quality Y2H library for vernalized plants of winter wheat. *Ta*VRN-A1 (VRN-A1 on chromosome 5A of *Triticum aestivum*) was used as a ‘bait’ to test the efficiency of the Y2H library. This study not only demonstrated the critical techniques to construct a high-quality Y2H library but also provided informative cues to unravel interacting proteins with *Ta*VRN-A1 involved in multiple developmental processes in wheat.

## Results and discussion

### Vernalized winter wheat plants used for the Y2H library

**Figure 1:**
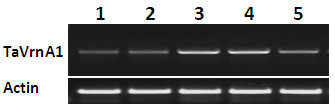
**Transcriptional levels of *TaVRN-A1 *in vernalized wheat cultivar 2174 using RT-PCR Lane 1: before vernalization; lanes 2–4: 1, 2, and 3 weeks of vernalization, respectively; lane 5: 1 week after vernalized plants was returned to the greenhouse; Actin acts as endogenous control.**

Leaves and shoot apexes are the main tissues to sense vernalization, and the transcripts of the vernalization genes mainly accumulate in leaves or/and shoot apexes in winter wheat [10]; hence, the two tissues were collected from winter wheat cv. 2174 plants that were treated for 1-3 weeks to ensure the sufficient pooling of genes responding to vernalization at early and late time in the Y2H library. To confirm that the sampled tissues were sufficiently vernalized, we tested the transcriptional change of *TaVRN-A1*, a key vernalization gene that was reported to have significant response to low temperature. RT-PCR showed that the transcript level of *TaVRN-A1* was gradually up-regulated in the winter wheat cultivar during vernalization (Figure 1), in the same pattern as previously reported [5,11-13]. This result indicated that total RNA isolated to construct Y2H library was representative of temporal and spatial expression patterns of vernalization-induced genes.

### Library quality

In addition to integrating wheat RNAs from shoot tissues sampled at different times during vernalization into our Y2H library, we also attempted to maximize absolute numbers of primary clones and average insert length, which are the important factors for the quality of a Y2H library [8,9]. To increase absolute numbers of primary clones, we not only got adequate ds cDNA substrate for transformation using SMART technology and high-quality LD-PCR but also performed high-efficiency transformation and *in vivo* recombination with the aid of commercial yeast transformation kit (≥3×10^5^ transformants/μg plasmid) and yeast native repair enzymes, respectively. To get enough long clone, we firstly used a high quality reverse transcriptase enzyme M-MLV, which can efficiently synthesize cDNA with long messenger RNA templates (>5 kb). Next, the PCR-enriched cDNA population (>200 bp) was recovered and purified from a low-melting gel. In fact, we also adopted Column Chromatography technology (CHROMA SPIN TE-400, Clontech, Cat. No. 636076) to perform cDNA size fractionation, but insert lengths in most of clones from the test Y2H library were considerably shorter than expected (not shown). Thus, the gel-based fractionation was used to get ds cDNA in our experiments, to supply high-quality cDNA for constructing Y2H library. This critical technique was used to remove the inhibitor for next steps, minimize truncated cDNAs, and eliminate primer dimers. After co-transformation of the fractionated ds cDNA and pGADT7-Rec into yeast AH109, the dilution test on SD/Leu media showed the transformation efficiency was 5.25×10^5^ cfu/μg AD vector.

**Figure 2:**
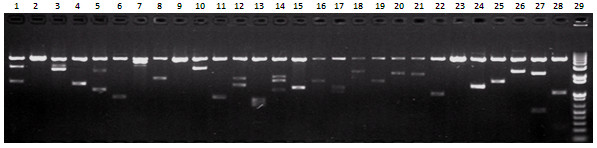
**The digestion identification for the insert sizes of Y2H library 1-28: The *Hind *III-digested AD fusion plasmids from the colonies randomly picked from Y2H library; 29: 1 Kb plus DNA ladder (Life technologies, Cat. No. 10787-018).**

In this study pGADT7-Rec of total 4.8 μg was used to construct the Y2H library, and 2.52×10^6^ primary clones were obtained. Hemacytometer counts showed the final concentration of the Y2H library was 2.6×10^8^ cells/ml, which far exceeded the minimum cell density, 2×10^7^ cells/ml, required for “mate-plate” Y2H screens. Meanwhile, we also checked the sizes of inserts in “prey” vectors using *Hind* III digestion (Figure 2). The average size of inserts in our Y2H library was about 1.5 kb (Additional file 1), which was comparable in insert size to the reported high quality *Brachypodium* Y2H library[9]. The quality values for our Y2H library are summarized in Table 1. In all, the Y2H library was expected to perform efficient Y2H screens.

**Table 1:** Measures of library quality

**Measured items**	**Values**
Transformation efficiency	5.25 × 10^5^ cfu/ μg AD vector
Total independent Colonies	2.52 × 10^6^ cfu/library
Library Concentration	2.6 × 10^8^ cells/ml
Minimum Length	0.13 kb
Maximum Length	5.2 kb
Average Length	1.544 kb

### Quality test of Y2H screens

Although the quality features of our Y2H library is good, most important of all is the effective identification of interacting proteins for a high-quality Y2H library. Thus we performed a proof-of-concept screen using selective “bait”. The ideal proteins for “bait” are those that have well-described interacting partners and can be used to quickly determine whether or not the expected interacting proteins can be captured. *Ta*VRN1 is MADS-Box type transcription factor (TF) and functions as the central regulator of vernalization pathway of flowering [14]. In addition, MADS-box proteins usually combine with other proteins to function in regulating gene expression [15]. Thus, it is very important to reveal interactome of *Ta*VRN1 with other proteins. Furthermore, previous study showed that *Ta*VRN1 interacted with proteins, such as wheat SOC1-like and SVP-like proteins identified in a conventional Y2H experiment in previous studies [16]. Among three homoeologous *TaVRN1* genes in hexaploid wheat, *TaVRN-A1* showed a strong genetic effect and a higher transcript level than *TaVRN-B1* and *TaVRN-D1*[17]. Therefore, we selected *Ta*VRN-A1 as “bait” to check the quality of theY2H library. In order to eliminate autoactivity of *Ta*VRN-A1 as ‘bait’ in Y2H system, we removed its MADs-box and C terminal, which are responsible to DNA binding and transcription initiation activity, respectively (Additional file 2).

Approximate 100 positive colonies were detected at QDO media and most of them were further identified on QDO/ X-α-Gal media (Additional file 3). Based on the difference of the X-α-Gal staining, 25 colonies were selected for further analysis. These clones were sequenced, resulting in 9 different genes (Table 2). Among them, a *Ta*SOC1-like protein (*Ta*SOC1=T. aestivum SUPPRESSOR OF OVEREXPRESSION CONSTANS 1) and two *Ta*SVP-like (*Ta*SVP=T. aestivum SHORT VEGETATIVE PHASE) were found. These two kinds of proteins were also detected as the partners of *Ta*VRN1 in conventional Y2H direct interaction assays [16].

**Table 2:** ***Ta*VRN-A1 unique interactors from Y2H screen**

**GenBank no.**	**Occurrence times**	**Name or homologue**
HX181945	3	Extended synaptotagmin-1-like protein (C2 superfamily)
AM502892	2	*Ta*SVP1-2B /*Ta*VRT2
CJ575323	1	CENP-E like kinetochore protein (KIP superfamily)
AM502888	6	*Ta*SOC1-3B
CD915716	3	DnaJ homolog subfamily B member (DnaJ_C superfamily)
AM502889	2	*Ta*SVP1-1A
EU081898	3	WubiG; Polyubiquitin
HX180757	4	Polyubiquitin (UBQ superfamily)
CA596538	1	Predicted RING-H2 finger protein

Note: the underlined appear at least twice in our Y2H screen. ‘A’ and ‘B’ for protein terms are clone names but not for chromosome names.

**Figure 3:**
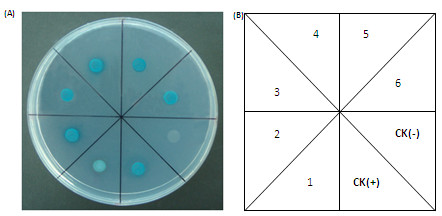
**The retest of the *Ta*VRN-A1 partners of interest.** X-α-Gal arrays for re-transformed Y2H cells on SD -Leu -Trp -His –Ade/ X-α-Gal media. CK (+): pGBKT7-53/SV40 T-antigen; CK (-): pGBKT7-Lam/SV40 T-antigen; 1: Polyubiquitin (WubiG); 2: Extended synaptotagmin-1-like protein; 3: *Ta*SVP-1A; 4: *Ta*SVP-2B/*Ta*VRT2; 5: *Ta*SOC1-3B; 6: DnaJ homolog subfamily B member. Note: the panels within the figure caption to correspond with the image.

To confirm interactions between *Ta*VRN-A1 and its partners of interest, we co-transformed the corresponding DBD and AD plasmids into yeast and re-identified on QDO/ X-α-Gal. The results confirmed these proteins had genuine interactions in living cells of yeast (Figure 3).

### *Ta*VRN-A1 might be an integrator in multiple developmental processes

This study revealed that *Ta*VRN-A1 could bind with quite a few of partners (Table 2), including two homoeologous SVP proteins, *Ta*SVP-2B and TaSVP-1A (=*Ta*VRT2) [18]. In addition, *Ta*VRN-A1 preferentially interacted with the *Ta*SOC1 and *Ta*SVP, which was observed at least two times. Their more frequent appearance among the positive clones indicated that the interactions among these proteins are not occasional but active in the context of the vernalized winter wheat plants. It has been reported that *Ta*VRT2 is a repressor [16]; whereas *Ta*SOC1 is an activator for flowering in plants [19]. All of *Ta*VRN1, *Ta*SVP and *Ta*SOC1 belong to MADS-type II protein and MADS-domain proteins often work in multimeric complexes with other MADS-domain proteins [20], due to the presence of the same structures (Additional file 2). The findings above allowed us to speculate that *Ta*VRT2 and *Ta*SOC1 might compete to interact with *Ta*VRN-A1 in the vernalization pathway of flowering.

In addition to the reported flowering-related partners, *Ta*VRN-A1 also showed interactions with the proteins involved in the other biological processes, including ubiquitin, synaptotagmin-1, and DnaJ (Table 2). It is well-known that ubiquitin tag not only can direct proteins to the proteasome for degradation of unneeded proteins but also can target proteins in the other biological process, such as DNA repair, autophagy and signal transduction [21]. It is likely that that *Ta*VRN-A1 is involved in ubiquitin-mediated protein degradation and cell regulation by interacting with ubiquitin. Previous studies showed that synaptotagmin-1 as a member of C2 superfamily could bind with Ca^2+^ and function as a Ca^2+^ sensor for spontaneous release [22], so it seemed that *Ta*VRN-A1 might be involved in Ca^2+^ regulation by interacting with Synaptotagmin-1. It is well-documented that DnaJ homolog subfamily B member (HSP40), a heat shock protein, acts as an important molecular chaperone responsive various types of environmental stress [23]. The interaction between *Ta*VRN-A1 and HSP40 observed in yeast suggested that *Ta*VRN-A1 might be related to regulation of multiple stresses through the heat shock protein pathway.

Altogether, the findings of the novel proteins searched with *Ta*VRN-A1 from our Y2H library screen provided informative cues for effects of *Ta*VRN-A1 on diverse biological processes in winter wheat.

## Conclusion

The availability of a high-quality Y2H library facilitates the constructing of the protein network stemming from a known protein. Notably, special attention should be paid to some critical aspects for high-efficiency Y2H screens. First, it is very important to select a suitable strategy to construct of Y2H library. It is more practical to construct a high-quality “narrow” Y2H library for a specific objective rather than a “broad” one for multiple purposes. Second, it is essential to make sure that experimental tissues are qualified for specific research objectives. The qualified tissues contain a high enrichment of the potential interactors so that it facilitates to discover the interaction of interest using the qualified tissues, especially in the developmental process with narrow time-window. Third, regardless of chromatography technology or gel-based recovery and purification, it was crucial to make a high-quality cDNA pool with a large range in size (>200 bp). Fourth, tens of putative positive colonies usually can be isolated from a normal-scale successful Y2H screen, but only some of them account for a high proportion of the total partners [8,9]. In this study, one quarter of total identified positive were selected for further analysis based on different interaction intensity with X-α-Gal staining. Sequencing analysis showed the reported partners of *Ta*VRN-A1 had been identified from the Y2H library and most of isolated clones appeared at least twice in the screen (Table 2). Considering yeast plasmid isolation and Plasmid Separation in Bacteria experiment are strenuous and time-consuming, grouping selection of positive clones based on their interaction strengths is a good knack to improve efficiency of Y2H screens. If you want get more complete information of the partners of the target protein, especially for its weak interaction partners, it had better analyze much more clones. Last, yeast mating for screening is of more easy-operation and high-efficiency compared to sequential transformation or co-transformation approach.

## Methods

### Plant sample preparation

The wheat cultivar ‘2174’ is a hard red winter wheat and it is an important source of several desirable traits [24,25]. 2174 was used throughout the experiments. The 3rd leaf stage seedlings were cold-treated at 4°C for 1- 3 weeks. Young leaves and shoot apexes were harvested from vernalized plants for different weeks and unvernalized plants used as control.

### RT- PCR

Total RNA was extracted using TRIzol® (Invitrogen) from mixed leaves and shoot apices in each sampling time-point, and were pretreated with recombinant RNase-free DNase I Nucleases (NEB, M0303) at 37°C for 30 minutes to eliminate contaminated genomic DNA. Reverse transcription was performed following the protocol of M-MLV (Promega, Cat. No. M1701). *TaVRN-A1* was used to test the quality of the cold-treated samples. Actin acted as internal control in this experiment. The primer sequences of *TaVRN-A1* and *Actin* were listed in Additional file 4.

### Construction of Y2H AD library and DBD fusion vector

The total RNA populations were first used to synthesize the first-strand using M-MLV (Promega, Cat. No. M1701) with CDS III Primer (specific primers for SMART™ technology) and then the resultant ss cDNAs serving as template were exponentially amplified by LD-PCR kit (Clontech, 2 PCR Kit, Cat. No. 639206) with the nested primers (5’ PCR primer and 3’ PCR primer) of SMART III™ (all primer sequences were provided in Additional file 4). The PCR products (>200 bp) were excised from 1% low melting Agarose gel and purified using Wizard® SV Gel and PCR Clean-Up System (Promega, Cat. No. A9281). The purified ds DNAs, together with linearized pGADT7-Rec AD cloning vector (Clontech, Cat. No. 630304), were co-transformed into yeast competent cell AH109, where yeast repair enzymes restore the linearized plasmid to its circular form by recombining homologous sequences at the end of the ds cDNA and pGADT7-Rec. This reaction was performed using Yeastmaker™ Yeast Transformation System 2 (Clontech Cat. No. 630439). In addition to culturing on SD/-Leu plates to select the transformants, a series of dilution of the transformed mixture also were spread on SD/Leu media to calculate the transformation efficiency and independent colonies. After culturing at 30°C for 6 days, the positive transformants were harvested to form an Y2H library. Hemacytometer was used to measure the cell density of the Y2H library to make sure the cell density ≥2 × 10^7^ cells/ml, which is essential for a high efficiency of the Y2H library screens. To exhibit the insert sizes of the ready Y2H library, the colonies were randomly picked out and their plasmids were isolated with user-developed protocol from Qiagen, and retransformed into *E*. *Coli* DH5α. Subsequently, the clones containing “prey” plasmids were obtained and their sizes were identified with *Hind* III digestion.

In this study, we selected *TaVrnA1* as “bait” to test the quality of the resultant Y2H library. Because its MADS domain is apt to cause auto-transcriptional activity in Y2H system, a truncated *TaVRN-A1* without the region encoding the MADS domain, *TaVRNA1*-ΔMADS, was generated with compatible restriction sites (*Nco* I and *Bam*H I) of pGBKT7 DBD (DNA binding domain) vector using the primers *TaVRNA1*-Y2H-F and *TaVRN-A1*-Y2H-R (Additional file 4). Digestion and ligation were performed to construct *TaVRN-A1*-ΔMADS DBD fusion vector. The integrity of the construct was verified by sequencing. Subsequently, the DBD fusion construct was transformed into the yeast strain Y187 and the positive colonies were selected on SD/-Trp medium. Furthermore, the toxicity and auto-transcriptional activation of the *TaVRN-A1*-ΔMADS BD construct were tested on SD/-Trp/ Kan (20 ug/ml) liquid medium and SD/-Ade/-Trp/-X-α-GAL plate, respectively.

### Screening Y2H AD library and analyzing positive interactions

The Y2H AD fusion library was screened using the corresponding BD fusion vector in yeast-mating way. The MATa library strain (e.g. AH109) with MATα bait-expressing reporter strain (e.g. Y187) were co-cultured, without time-consuming and labor-intensive plasmid extraction as well as co-transformation steps required in traditional Y2H screens. The 5 ml Y187 containing bait construct, pGBKT7-TaVRNA1-ΔMADS, was combined with 1 ml original Y2H library (hosted in AH109) in a 2 L flask. The mating-resultant zygotes cells were spread on QDO to select for putative positive two-hybrid interactions. We selected those that grew normally 3-8 days after plating in selective medium based on color (white or light pink) and growth speed (can grow to >2 mm). Next, the putative positive colonies were streaked out on master plate with QDO/ X-α-Gal for further identification and interaction intensity measurement. The positive yeast colonies with different interaction intensity were grouped and selected for further analysis. The AD plasmid of the selective colonies were rescued by Plasmid Segregation in Bacteria experiments and then identified by sequencing. In order to confirm the protein-protein interactions of interest, the target plasmid combinations were co-transformed into yeast AH109 and retested on QDO/ X-α-Gal media.

## Abbreviations

RT-PCR: Reverse transcription PCR; SMART™: Switching Mechanism At 5' end of RNA Transcript; AD: Activated domain; DBD: DNA binding domain; LD-PCR: Long distance PCR; SD: Synthetic defined media; QDO: Quadruple Dropout Medium: SD/-Ade/-His/-Leu/-Trp

## Competing interest

The authors declare that they have no competing interest.

## Authors’ contributions

SC preformed all experimental procedures, LY supplied critical reviews and very helpful suggestions for the manuscript. Both authors read and approved the final manuscript.

## Supplementary Material

Additional file 1 Insert sizes of the colonies randomly picked from the resultant wheat vernalizatioin library. Click here for file

Additional file 2 **The schematic diagram of MADS-Box proteins This diagram is based on the review of [**[26]**].** Click here for file

Additional file 3 ** pGBKT7-53/SV40 T-antigen; CK (-): pGBKT7-Lam/SV40 T-antigen.** The rest stripy colonies were directly picked from the original QDO plates of Y2H screen. Click here for file

Additional file 4 Primer list in this research. Click here for file
