# Supplementary material for: Construction of a high-quality yeast two-hybrid (Y2H) library and its application in identification of interacting proteins with key vernalization regulator TaVRN-A1 in wheat
Source: BMC Res Notes. 2013 Mar 5;6:81. doi: 10.1186/1756-0500-6-81 (PMC3605349; doi:10.1186/1756-0500-6-81)
Supplement: Additional file 3 — pGBKT7-53/SV40 T-antigen; CK (-): pGBKT7-Lam/SV40 T-antigen. The rest stripy colonies were directly picked from the original QDO plates of Y2H screen. [file 1756-0500-6-81-S3.docx]

**CK(-)**

**CK(+)**

**CK(-)**

**CK(+)**
