# Supplementary material for: Construction of a high-quality yeast two-hybrid (Y2H) library and its application in identification of interacting proteins with key vernalization regulator TaVRN-A1 in wheat
Source: BMC Res Notes. 2013 Mar 5;6:81. doi: 10.1186/1756-0500-6-81 (PMC3605349; doi:10.1186/1756-0500-6-81)
Supplement: Supplementary file 3 [file 1756-0500-6-81-S2.docx]

I region

MADS-box

K-box

C terminal

DNA binding 

and dimerization

protein-protein interaction

(heterodimerization)

transcriptional activation activity

homodimerization functional specificity
